# Very late-onset endocarditis complicated by non-significant aortic regurgitation after device closure of perimembranous ventricular septal defect

**DOI:** 10.1097/MD.0000000000020120

**Published:** 2020-05-08

**Authors:** Changqing Tang, Kaiyu Zhou, Yimin Hua, Chuan Wang

**Affiliations:** a Department of Pediatric Cardiology; b The Cardiac Development and Early Intervention Unit, West China Institute of Women and Children's Health, West China Second University Hospital, Sichuan University; c Key Laboratory of Birth Defects and Related Diseases of Women and Children (Sichuan University), Ministry of Education; d Key Laboratory of Development and Diseases of Women and Children of Sichuan Province, West China Second University Hospital, Sichuan University, Chengdu, Sichuan, China.

**Keywords:** aortic valve regurgitation, infective endocarditis, perimembranous ventricular septal defect

## Abstract

**Introduction:**

Aortic regurgitation (AR) was recognized as a major, but rare complication after device closure for perimembranous ventricular septal defects (PmVSD). Most of them are temporary and non-significant. Infectious endocarditis (IE) is another extremely rare post-procedure complication of PmVSD. Theoretically, AR could increase risk for post-interventional IE. However, no cases have been documented thus far. We firstly described a case of very late-onset IE associated with non-significant AR after transcatheter closure of PmVSD with modified symmetrical double-disk device, underscoring the need for reassessing long-term prognostic implications of non-significant post-procedure AR after PmVSD occlusion and the most appropriate treatment strategy.

**Patient concerns:**

A 15-year old male received transcatheter closure of a 6.4 mm sized PmVSD with a 9-mm modified symmetric double-disk occluder (SHAMA) 11 years ago in our hospital. A new-onset mild eccentric AR was noted on transthoracic echocardiography (TTE) examination 1-year post procedure, without progression and heart enlargement. At this time, the child was admitted with a complaint of persistent fever for 16 days and nonresponse to 2-weeks course of amoxicillin and cefoxitin.

**Diagnosis:**

The diagnosis of post-procedure IE was established since a vegetation (14 × 4 mm) was found to be attached to the tricuspid valve, an anechoic area (8 × 7 mm) on left upper side of ventricular septum and below right aortic sinus, and severe eccentric AR as well as the isolation of *Staphylococcus aureus* from all three-blood cultures.

**Interventions:**

Treatment with vancomycin was initially adopted. However, surgical interventions including removal of vegetation, abscess and occluder, closure of VSD with a pericardial patch, tricuspid valvuloplasty, and aortic valvuloplasty were ultimately performed because of recurrent fever and a new-onset complete atrioventricular block 12-days later. The child continued with antibiotic therapy up to six weeks post operation.

**Outcomes:**

The child's temperature gradually returned to normal with alleviation of AR (mild) and heart block (first degree). The following course was uneventful.

**Conclusion:**

Late-onset IE could occur following device closure of PmVSD and be associated with post-procedure AR. For non-significant AR after device closure of PmVSD, early surgical intervention could be an alternative for reducing the aggravation of aortic valve damage and the risk of associated IE.

## 1 Introduction

Transcatheter closure of perimembranous ventricular septal defect (PmVSD) has been proved to be a safe and effective alternative to surgery in selected patients.^[1,2]^ Aortic regurgitation (AR), attributable to impingement of device on aortic valve, was recognized as a major, but rare complication after device closure for PmVSD. Most of them occurred early and a high proportion is temporary and non-significant.^[1]^ Despite presence of a foreign body, post-procedure infectious endocarditis (IE) is considered an extremely rare complication following device closure of PmVSD. Up to now, only two cases have been reported.^[3,4]^ All of them occurred early after procedure and are speculated resulting from introduction of microbes during implantation, incomplete device endothelialization or residual shunt.^[3,4]^ Theoretically, AR could increase risk for post-interventional IE. No instances, however, have been reported. Herein, We firstly described a case of very late-onset IE complicated by AR occurring 11 years following device closure of PmVSD with modified symmetrical double-disk device, underscoring importance and necessity of long-term, perhaps life-long follow-up for these patients, and illustrates need for reassessing long-term prognostic implications of non-significant post-procedure AR after device closure of PmVSD and the most appropriate treatment strategy.

## 2 Ethics statements

Informed written consent was obtained from the parents after the nature of this study had been fully explained to them. The parents of patient have provided informed consent for publication of the case.

## 3 Case report

**Figure 1:**
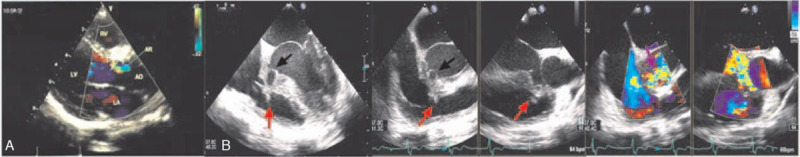
(A) Parasternal long-axis views on TTE 10 years post device closure showed mild eccentric AR. (B) Short-axis views on TEE 11 years post device closure revealed vegetation attached to TV (red arrow), an anechoic area below right aortic sinus (black arrow), continuity between aorta and anechoic area (purple arrow) and severe eccentric AR. AR = aortic regurgitation, TEE = transesophageal echocardiography, TTE = transthoracic echocardiography.

A 15-year old male was admitted into emergency department of our hospital in January 2019 due to persistent fever for 16 days and nonresponse to 2-weeks course of amoxicillin and cefoxitin. The child had received transcatheter closure of a 6.4 mm sized PmVSD with a 9-mm modified symmetric double-disk occluder (SHAMA) 11 years ago in our hospital. The procedure was undertaken under general anesthesia and performed in a standard way detailed in our previous study.^[5]^ The device was released without any complications such as residual shunt and AR. Oral administration of aspirin (3–5 mg/kg daily) was initiated for 6 months and the child was subjected to 72 h of dynamic electrocardiogram (ECG) monitoring, as well as a 12-lead ECG and echocardiography at 1, 3, and 7 days post procedure. After discharge, he was followed up clinically as well as with 24-h dynamic ECG, and echocardiography at 1, 3, 6, and 12 months during the first year and annually thereafter. A new-onset mild eccentric AR was noted on transthoracic echocardiography (TTE) examination 1-year post procedure, without progression and left ventricular enlargement until latest follow-up in January 2018 (Fig. 1A).

On admission, the child was conscious with temperature of 39°C, heart rate of 78 per minute, respiratory rate was 22 per minute, and blood pressure was 114/53 mm Hg. Physical examination was only remarkable for a diastolic murmur in auscultation area of aortic valve. Laboratory tests revealed an elevated white blood cell count of 15.89 × 109/L, neutrophil percentage of 80.1%, platelet count of 527 × 109/L, C-reactive protein of 36.3 mg/L, erythrocyte sedimentation rate of 62.0 mm/h, and mild anemia (hemoglobin: 116 g/L). Transesophageal echocardiography (TEE) demonstrated a vegetation (14 × 4 mm) attached to tricuspid valve (TV), an anechoic area (8 × 7 mm) on left upper side of ventricular septum and below right aortic sinus, and severe eccentric AR. The diagnosis of IE was established. Three sets of blood cultures were taken and empiric antibiotic therapy consisting of vancomycin and ceftriaxone was initiated. *Staphylococcus aureus* was isolated from all three-blood cultures. Treatment with vancomycin was continued since the isolates were resistant to penicillin but sensitive to vancomycin. The origin of this infection was not found. Results of a dental checkup performed during hospitalization were normal. The patient ultimately underwent an emergent surgical intervention because of recurrent fever and a new-onset complete atrioventricular block 12-days later. During the procedure, contracture of right coronary valve, fusion of upper margin of occluder with right coronary valve, perforation of interventricular septal abscess below right coronary valve and vegetation on TV were documented. Removal of vegetation, abscess and occluder, closure of VSD with a pericardial patch, tricuspid valvuloplasty and aortic valvuloplasty were performed. The child continued with antibiotic therapy up to six weeks post operation. The temperature gradually returned to normal with alleviation of AR (mild) and heart block (first degree). The following course was uneventful.

## 4 Discussion

Even though native VSD endocarditis is well known,^[6]^ device-associated endocarditis following PmVSD closure is scarce. Up to now, only two cases with post procedure IE occurred 10 days^[4]^ and 4 months^[3]^ after transcatheter closure of PmVSD with Nit-Occlud Le device, respectively, have been reported. Both of them were not associated with post-procedure AR. To the best of our knowledge, this is the first case of IE complicated by AR following percutaneous PmVSD occlusion with modified symmetric double-disk occluder. A recent meta-analysis reported 106 patients across 34 studies presented with AR after device closure of PmVSD, with a pooled incidence of 2.0%.^[1]^ Given the worldwide acceptance and increasing utilization of device closure for PmVSD, there are likely additional patients with delayed incorporation that could present a late risk for AR and IE. This study was of clinically amount significance for illustrating the requirement of re-estimating the prognostic implication of non-significant AR after PmVSD device closure and taking the most reasonable deals into consideration for this complication.

Actually, apart from severe arrhythmias including high degree or complete atrioventricular block and complete left bundle branch block, post-procedure AR is always another major issue of concern following device closure of PmVSD due to the possibilities of occluder impingement on aortic valve. However, most of the AR was clinically non-significant and may not lead to heart enlargement. For the non-significant AR following device closure of PmVSD, although lifelong antibiotic prophylaxis has been recommended for any type of congenital heart defect repaired with a prosthetic material, whether placed surgically or by percutaneous techniques if valvular regurgitation remains,^[7,8]^ few followed the current guidelines^[9]^ and regular follow-up were chosen for these patients by most clinicians since the risk of antibiotic-associated adverse events may outweigh the benefits and most importantly, IE associated with AR after device closure of PmVSD has never been reported before.

Our case suggested that early surgical intervention could be alternatively considered even for non-significant AR and it may be a better and more satisfactory means for reducing the aggravation of aortic valve damage and the risk of associated IE.

In conclusion, the development of AR resulted from impingement of occluder on aortic valve and associated late-onset IE after device closure of PmVSD in this patient are both causes for concern. For non-significant AR after device closure of PmVSD, early surgical intervention may be a better alternative compared with lifelong antibiotic prophylaxis or no interventions, since associated IE can indeed occur despite its rarity and the risk of antibiotic-associated adverse events may outweigh the benefits.

## Author contributions

**Resources:** Kaiyu Zhou, Yimin Hua, Chuan Wang.

**Supervision:** Kaiyu Zhou, Yimin Hua, Chuan Wang.

**Writing – original draft:** Changqing Tang.

**Writing – review & editing:** Changqing Tang, Kaiyu Zhou, Chuan Wang.
